# Characterization of Influenza B Virus Variants with Reduced Neuraminidase Inhibitor Susceptibility

**DOI:** 10.1128/AAC.01081-18

**Published:** 2018-10-24

**Authors:** R. Farrukee, A. E. Zarebski, J. M. McCaw, J. D. Bloom, P. C. Reading, A. C. Hurt

**Affiliations:** a WHO Collaborating Centre for Reference and Research on Influenza, Peter Doherty Institute for Infection and Immunity, Melbourne, Victoria, Australia; b Department of Microbiology and Immunology, The University of Melbourne, Peter Doherty Institute for Infection and Immunity, Melbourne, Victoria, Australia; c School of Mathematics and Statistics, The University of Melbourne, Melbourne, Australia; d Centre for Epidemiology and Biostatistics, Melbourne School of Population and Global Health, The University of Melbourne, Melbourne, Australia; e Victorian Infectious Diseases Reference Laboratory Epidemiology Unit, Peter Doherty Institute for Infection and Immunity, Melbourne, Victoria, Australia; f Infection and Immunity theme, Murdoch Children's Research Institute, Royal Children's Hospital, Melbourne, Australia; g Division of Basic Sciences and Computational Biology Program, Fred Hutchinson Cancer Research Center, Seattle, Washington, USA

**Keywords:** influenza B, neuraminidase inhibitor, resistance, viral fitness

## Abstract

Treatment options for influenza B virus infections are limited to neuraminidase inhibitors (NAIs), which block the neuraminidase (NA) glycoprotein on the virion surface. The development of NAI resistance would therefore result in a loss of antiviral treatment options for influenza B virus infections. This study characterized two contemporary influenza B viruses with known resistance-conferring NA amino acid substitutions, D197N and H273Y, detected during routine surveillance. The D197N and H273Y variants were characterized *in vitro* by assessing NA enzyme activity and affinity, as well as replication in cell culture compared to those of NAI-sensitive wild-type viruses. *In vivo* studies were also performed in ferrets to assess the replication and transmissibility of each variant. Mathematical models were used to analyze within-host and between-host fitness of variants relative to wild-type viruses. The data revealed that the H273Y variant had NA enzyme function similar to that of its wild type but had slightly reduced replication and transmission efficiency *in vivo*. The D197N variant had impaired NA enzyme function, but there was no evidence of reduction in replication or transmission efficiency in ferrets. Our data suggest that the influenza B virus variant with the H273Y NA substitution had a more notable reduction in fitness compared to wild-type viruses than the influenza B variant with the D197N NA substitution. Although a D197N variant is yet to become widespread, it is the most commonly detected NAI-resistant influenza B virus in surveillance studies. Our results highlight the need to carefully monitor circulating viruses for the spread of influenza B viruses with the D197N NA substitution.

## Abstract (precis)

Treatment options for influenza B virus infections are limited to neuraminidase inhibitors (NAIs), which block the neuraminidase (NA) glycoprotein on the virion surface. The development of NAI resistance would therefore result in a loss of antiviral treatment options for influenza B virus infections.

## INTRODUCTION

Influenza A and B viruses cocirculate in the human population and cause yearly epidemics worldwide. Even though influenza B viruses generally cause a milder disease than influenza A viruses, in certain seasons, they can contribute to a substantial burden of disease and up to 25% of influenza-related mortality (1, 2). Vaccines are the main control measure for influenza disease prevention, but a recent analysis of data between 2004 and 2014 revealed that the effectiveness of the vaccine against influenza B virus infections was only 54% (3).

The neuraminidase inhibitors (NAIs) (zanamivir, oseltamivir, laninamivir, and peramivir) are a class of antiviral drugs that target the conserved amino acid residues of the viral neuraminidase (NA) active site and competitively inhibit enzyme function (4, 5). However, mutations in the NA enzyme active site or adjacent framework residues can abrogate NAI interaction with the NA and reduce the susceptibility of viruses to one or more of these drugs (6). Antiviral options for the treatment of influenza B viruses are limited to NAIs, as the older class of influenza antivirals, the adamantanes, are only effective against influenza A viruses (6). Development of NAI resistance in influenza B viruses is therefore a public health concern, as it would remove a valuable treatment option.

*In vitro* studies consistently demonstrate that compared to influenza A viruses, influenza B viruses exhibit reduced sensitivity to oseltamivir, suggesting that the drug may already have reduced effectiveness against influenza B viruses (6–10). The clinical relevance of this has not been fully elucidated, but in 7 out of 9 clinical studies, it was shown that oseltamivir treatment resolved symptoms faster in influenza A virus patients than in influenza B virus patients (11). Considering this, it is possible that NA mutations that only moderately alter the oseltamivir susceptibility of influenza B viruses may have a significant impact on the clinical effectiveness of the drug.

A number of different NA substitutions at conserved amino acid positions (e.g., E117, D197, I221, and H273) have previously been described to confer reduced inhibition by the NAIs *in vitro* (8, 12–21), but the impact of these substitutions on enzyme function, virus replication, or transmissibility has only been assessed in a limited number of studies (14, 22, 23). The fitness of influenza B viruses with either the H273Y or D197N NA substitution is of particular interest, as a number of viruses with either substitution have been recently found in patients in community settings who, unlike hospitalized or immunocompromised patients, do not typically receive NAI treatment (8, 9, 17, 18, 24). Two reports have identified household transmission of influenza B viruses with the D197N NA substitution (18, 25), and more recently, a global surveillance report identified a cluster of six influenza B viruses with the D197N NA substitution in Japan in early 2014, further suggesting potential community transmission of the variant virus (18). Interestingly, 22 out of the 27 viruses with the D197N substitution reported in the literature were from the B/Yamagata lineage (17, 18, 25–30). There have also been examples of suspected transmission of influenza B viruses with the H273Y NA substitution (9). The H273Y NA substitution in influenza B viruses occurs at the equivalent residue to that of the H275Y NA substitution in influenza A(H1N1) viruses, which was present in the oseltamivir-resistant influenza A(H1N1) viruses that spread globally in 2008/2009 (31, 32).

The effect of H273Y NA substitutions in influenza B viruses has been previously studied using reverse genetics (rg) in the B/Yamanashi/166/98 virus background (15, 22, 23). To date, few studies have reported the effect of the H273Y or the D197N NA substitution on contemporary viruses, which is important because it has been shown that the fitness consequences of resistance-conferring mutations can vary due to the genetic background of the NA (33, 34). Although experiments using reverse genetics can be useful in defining the impact of a single mutation on viral fitness, they do not evaluate the effect of the rest of the viral genome that may play an important role in the fitness of that virus. Our aim was to characterize two “naturally” occurring influenza B variant viruses containing either the H273Y or D197N NA substitution which had been detected during routine surveillance in patients not being treated with NAIs, compared to closely matched wild-type viruses by assessing their enzyme function, *in vitro* replication, and *in vivo* replication and transmission.

## RESULTS

### NAI susceptibility, NA activity, surface expression, and substrate affinity.

**FIG 1:**
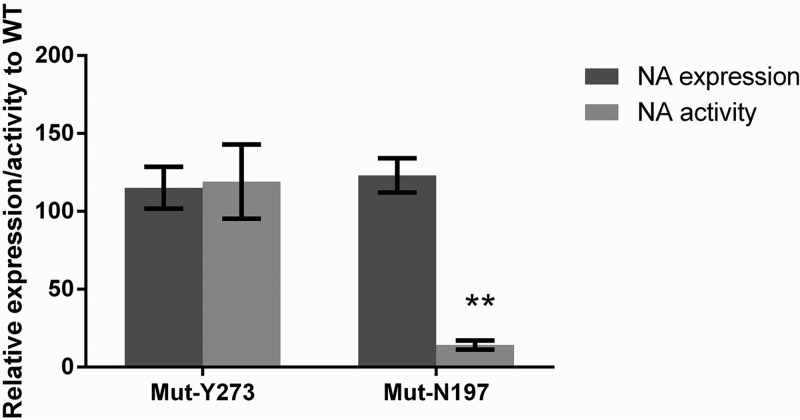
The mean NA surface expression and activity of influenza B variants relative to the corresponding WT. HEK-293T cells were transfected with plasmids containing the NA gene encoding WT and variant proteins. At 20 h posttransfection, cells were analyzed for NA activity using a MUNANA-based assay and for NA expression using a fluorescence-activated cell sorter (FACS)-based assay. The activity and expression data of the variants were normalized relative to their corresponding WT and expressed as the mean ± standard error of the mean (SEM). Data are derived from two independent experiments, each containing triplicate samples, and were compared between relevant groups using the nonparametric Mann-Whitney U test.

The effects of the D197N and H273Y substitutions on NA enzyme function were assessed using four different assays. The mutant Y273 (MUT-Y273) variant had a 3-fold increase in oseltamivir 50% inhibitory concentration (IC_50_) and an 85-fold increase in peramivir IC_50_ compared to the wild-type H273 virus (WT-H273), but the IC_50_s for zanamivir and laninamivir were not significantly different (Table 1). The MUT-Y273 virus had comparable *K_m_* (substrate affinity) to that of the WT-H273 virus (Table 1). Similarly, the relative NA surface expression and enzyme activity of the MUT-Y273 virus compared to the WT-H273 virus were 115% ± 13.4% (mean ± standard error of the mean [SEM]) and 119% ± 23.1%, respectively, neither of which was significantly different (Fig. 1).

**TABLE 1:** Effect of neuraminidase mutation on IC_50_ and enzyme kinetics

Virus	Mean ± SD (fold difference)^*a*^				
	IC_50_ (nM) for:				*K_m_* (μM)
	Zanamivir	Oseltamivir	Laninamivir	Peramivir	
WT-H273	2.2 ± 1.3	36.1 ± 5.4	2.6 ± 2.0	1.0 ± 0.05	11.5 ± 1.5
Mut-Y273	1.4 ± 0.5 (1)	96.7 ± 10.8 (3)	1.63 ± 0.4 (1)	80.9 ± 6.8 (85)^*b*^	12.3 ± 0.8 (1)
WT-D197	1.6 ± 0.2	35.1 ± 3.3	2.8 ± 1.3	1.0 ± 0.1	13.8 ± 1.3
Mut-N197	4.5 ± 0.7 (3)	66.2 ± 7.5 (2)	5.0 ± 1.2 (2)	3.0 ± 0.7 (3)	30.3 ± 3.3 (3)

a Fold difference is from corresponding wild-type values. The data are from three independent experiments.

b MUT-Y273 has a significantly higher IC_50_ for peramivir than that of WT-H273 (Mann-Whitney's U test, *P* < 0.05).

The MUT-N197 virus had a 2- to 3-fold reduction in susceptibility to all four NAIs compared to that of the WT-D197 virus (Table 1). The substrate affinity (*K_m_*) was 3-fold lower for the MUT-N197 virus than for the WT-D197 virus (*K_m_* values are inversely proportional to substrate affinity), but this difference was not statistically significant (Table 1). The cell surface expression of the NA enzyme of the MUT-N197 variant was 123% ± 10.9% relative to the WT-D197 virus, but the NA enzyme activity of the MUT-N197 variant was only 14.2% ± 3.0%, which was significantly lower (*P* < 0.01) than that of the WT-D197 virus (Fig. 1).

### *In vitro* replication kinetics.

**FIG 2:**
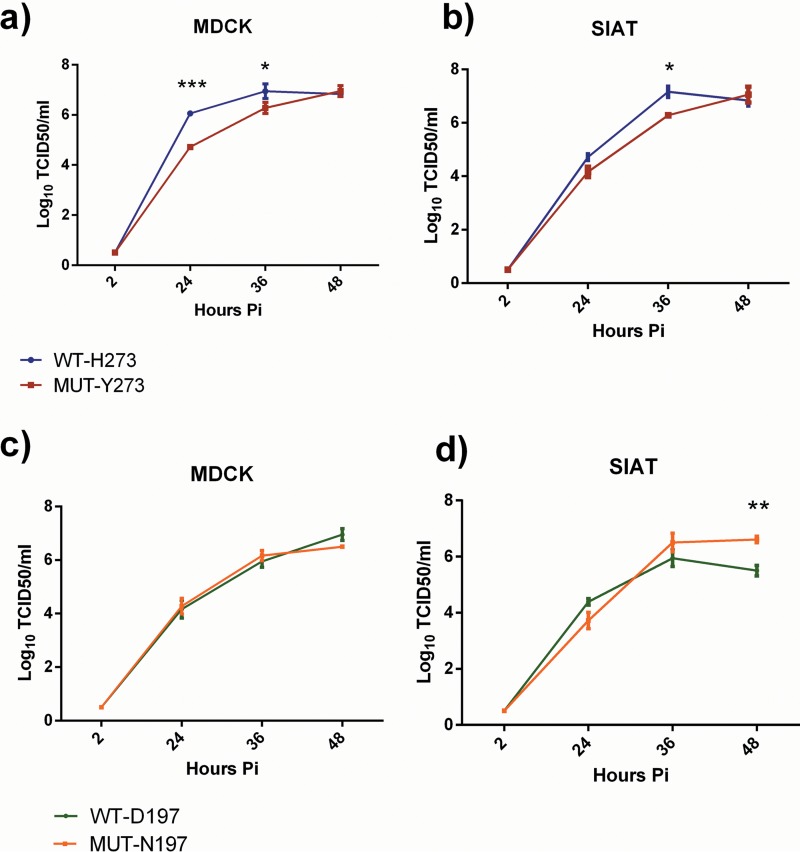
Multicycle replication of influenza B WT and MUT viruses in MDCK and SIAT cells. Cells were infected at an MOI of 0.01 and supernatants collected and assayed for infectious virus at 2, 24, 36, and 48 h postinfection. Data show the mean (± SEM) of triplicate samples for MUT-Y273 versus WT-H273 (a and b) and MUT-N197 versus WT-D197 (c and d). A two-way ANOVA with Bonferroni's posttest analysis was done to compare mean viral titers at each time point between WT and MUT viruses. *, *P* < 0.05; **, *P* < 0.01; ***, *P* < 0.001.

Multistep growth curves were performed in MDCK and MDCK-SIAT (SIAT) cells to assess the *in vitro* replication kinetics of the WT and MUT viruses. The MUT-Y273 variant showed significantly reduced viral titers compared to the WT-H273 virus at 24 and 36 h postinfection in MDCK cells and at 36 h postinfection in SIAT cells (Fig. 2a and b). However, at 48 h postinfection, there were no significant differences in viral titers between the MUT-Y273 and WT-H273 viruses in either cell line.

The MUT-N197 variant demonstrated no reduction in replication compared to the WT virus in MDCK cells and actually outgrew the WT-D197 virus in SIAT cells at 48 h postinfection, where it reached a significantly higher titer of 6.61 ± 0.11 versus 5.5 ± 0.2 log_10_ 50% tissue culture infective dose (TCID_50_)/ml (*P* < 0.01) (Fig. 2c and d).

### Replication and transmission of pure viral populations in ferrets.

**FIG 3:**
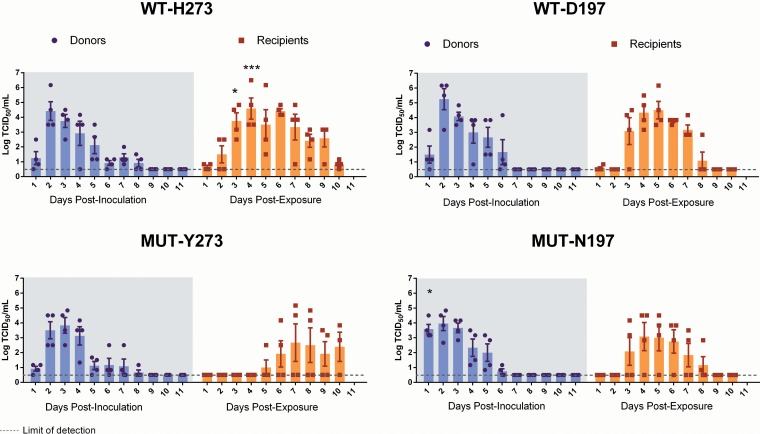
Viral shedding in nasal wash samples from donor and recipient ferrets infected with WT or MUT influenza B viruses. Donor ferrets (*n* = 4 per group) were experimentally infected with 10^5^ TCID_50_ of pure WT or MUT virus. Naive recipients were cohoused with each donor 1 day after experimental infection of the donors. Daily nasal wash samples were collected from both donors and recipients over a period of 11 days, and titers of infectious virus in nasal wash samples were determined using a TCID_50_ assay. (A to D) Summary of TCID_50_ results, with the bar graphs showing the mean (± SEM) titers at each day for each group. At each time point, mean viral titers were compared between donors infected with either WT or MUT virus by two-way ANOVA with Bonferroni's posttest analysis. Similarly, analysis was done to compare titers between recipients infected with either WT or MUT virus. Significant differences in mean viral titers were observed between WT-H273- and MUT-Y273-infected recipients on day 3 and 4 postexposure and between WT-D197- and MUT-N197-infected donors on day 1 postinoculation. The AUCs of the TCID_50_ graphs were also calculated and compared by Mann-Whitney's U test; no significant differences were detected between donor animals infected with WT and corresponding MUT viruses. *, *P* < 0.05; **, *P* < 0.01; ***, *P* < 0.001.

To gain insight regarding virus replication *in vivo*, we determined titers of infectious virus in nasal wash samples taken at various times after intranasal infection of ferrets. Figure 3a and c summarizes the results for animals infected with either the WT-H273 or the MUT-Y273 virus. In both donor groups (infected by direct intranasal inoculation), infectious virus was detectable until day 8 postinfection, and peak viral titers were not significantly different. There was also no significant difference in the area under the concentration-time curve (AUC) values of TCID_50_ plots over the 10-day period (Fig. 3a and c). Body temperature and weight were also assessed daily after infection, and no significant differences were noted between donor animals infected with WT-H273 or MUT-Y273, although there was a trend toward greater weight loss in donors infected with WT-H273 (Fig. S2).

We examined virus replication in nasal washes from naive recipient ferrets who were cohoused with experimentally infected donors to assess the transmissibility of WT-H273 and MUT-Y273 viruses. All four recipients cohoused with WT-H273-infected donors shed infectious virus, but only two out of four recipients cohoused with MUT-Y273-infected donors shed detectable infectious virus (Fig. 3a and c). There was a delay in transmission of the MUT-Y273 virus compared to the WT-273 virus, as peak virus titers in WT-H273-infected recipients were detected on day 4 postexposure, while the two recipients infected with the MUT-Y273 virus reached peak viral titers on day 7 postexposure (Fig. 3a and c).

Next, we examined virus replication following experimental infection of animals with WT-D197 and MUT-N197 viruses. The kinetics of viral shedding was similar between donor ferrets experimentally infected with either WT-D197 or MUT-N197 virus (Fig. 3b and d). The AUCs of the viral replication plots and the peak viral titers between the donor groups were not significantly different. Infectious viral titers were detectable in nasal washes of at least one ferret until day 6 postinfection, and donors did not experience any significant changes in either body weight or temperature (Fig. S2).

The WT-D197 virus transmitted successfully to all four recipients, while the MUT-N197 virus transmitted to three out of the four recipients. However, durations of viral shedding and the peak viral titers were not significantly different between recipients infected with either the WT-D197 virus or the MUT-N197 virus (Fig. 3b and d); there was also no transmission delay observed as with MUT-Y273. Similar to the donor ferrets, the recipient ferrets infected with either WT-D197 or MUT-N197 virus experienced no significant changes in either body weight or temperature.

Pyrosequencing analysis of relevant NA substitutions in nasal wash samples from all infected animals confirmed that neither the D197N nor the H273Y substitution was lost following replication within the airways of donor ferrets, or following transmission to recipient animals. Next-generation sequence analysis on nasal wash samples from the last day of shedding in recipient ferrets was performed to confirm the genetic stability of the rest of the viral genome upon transmission. In addition to a number of synonymous mutations found across the influenza genome, the MUT-Y273 virus from one recipient ferret also contained a M403V NA amino acid substitution, and the WT-D197 virus from all recipients contained an L274I amino acid substitution in the PA enzyme. M403V in influenza B NA is distant from the enzyme active site and has only occurred on rare occasions in influenza B viruses (19 out of 5,556 sequences in the Global Initiative on Sharing All Influenza Data [GISAID]), while the PA substitution L274I has not been previously observed in any of the influenza B PA gene sequences on GISAID.

### Relative fitness between viral pairs based on “competitive-mixtures” analyses.

To gain further insight into the fitness differential between each variant virus and their corresponding wild-type viruses, a competitive-mixtures experiment was performed in ferrets. Mathematical models for the progression of the infection and its transmission were fitted to measurements collected during the experiment. The parameter estimates from this fitting process provide information about the relative fitness of the variant for replication within a host and its ability to transmit between hosts.

**FIG 4:**
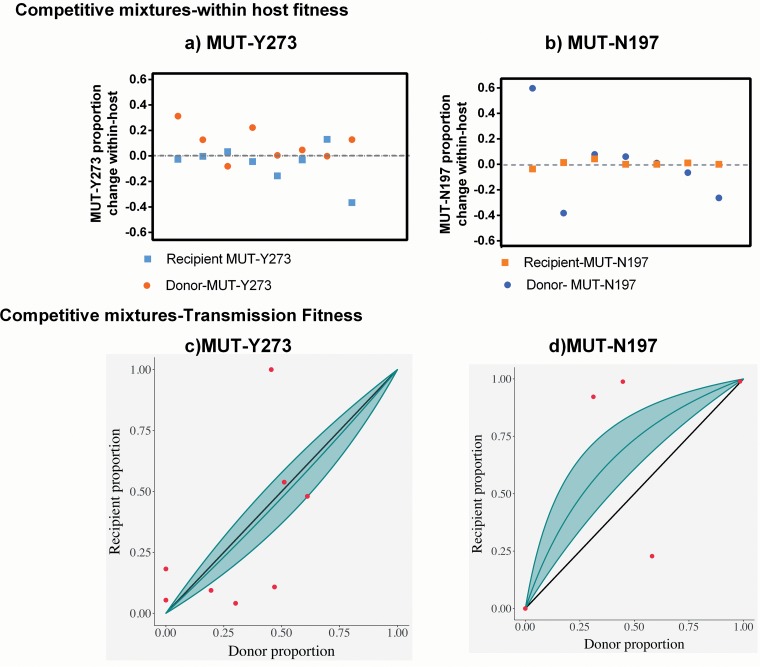
Summary of changes in mutant virus proportions in nasal washes within-host and during transmission in competitive mixture ferret experiments. To assess within-host and transmission fitness of mutant viruses relative to their corresponding wild types, ferrets were experimentally coinfected with mixtures of WT and MUT viruses at different ratios (50:50, 80:20, and 20:80) and cohoused with naive recipients. The proportion of WT versus MUT was determined in the collected nasal wash samples by pyrosequencing, and data were analyzed only from animals where the proportion of WT and MUT viruses could be accurately determined. (a and b) Individual points that correspond to the change in the proportion of mutant virus in one ferret between the first day of infection and last day of infection (*y* axis). This allows for a visual representation of within-host fitness. (c and d) Individual points that correspond to a transmission event between two ferrets. In these plots, the abscissa is the proportion of MUT virus in donor ferrets the day preceding confirmed transmission, and the ordinate shows the proportion of MUT virus in recipient ferrets within the first 24 h of confirmed infection. The solid gray line is the representative curve of the shape parameter, calculated by fitting our data to a model described in more detail in Materials and Methods and the supplemental material. The 95% confidence interval is represented by the shaded region. If our fitted model lies above the symmetry line (*y* = *x* solid line), the mutant virus is fitter than the wild-type virus, whereas if the fitted model lies below the symmetry line, the converse is true.

These inferences are summarized in Table 2, and visualizations are shown in Fig. 4.

**TABLE 2:** Relative within-host and transmission fitness between viral pairs tested in competitive mixtures during ferret infection

Mutant	Corresponding wild type	Relative within-host fitness (*R*_0_ ratio [95% CI])^*a*^	Relative transmission fitness (95% CI)^*b*^
Mut-Y273	WT-H273	0.84 (0.70, 1.02)	0.90 (0.60, 1.35)
Mut-N197	WT-D197	0.94 (0.81, 1.10)	2.78 (1.56, 5.53)

a If the *R*_0_ ratio is <1, it indicates fitness advantage of wild type over the mutant, and if the *R*_0_ ratio is >1, the mutant is fitter than the wild type.

b Relative transmission fitness value of <1 indicates fitness advantage of the wild type over mutant, and a fitness value of >1 indicates that the mutant is fitter than the wild type.

We define the relative within-host fitness as the ratio (*R*_0_) of the basic reproduction numbers of the variant compared to its wild-type counterpart. For the MUT-Y273 virus, the *R*_0_ ratio is estimated to be 0.84 with a 2-sided 95% confidence interval (CI) of 0.70, 1.02, with our analysis indicating that there is a 95% chance that this ratio is less than 1. So while the MUT-Y273 variant is almost certainly less fit than its wild-type counterpart, the difference is very small. There was little evidence that the mutation affected transmission fitness, with the mutant's ability to transmit reduced by a factor of 0.90 (95% CI, 0.60, 1.35).

For MUT-D197, we find little evidence for a difference in within-host fitness, with an estimated ratio of 0.94 (95% CI, 0.81, 1.10) and a 75% chance that the variant is less fit than the wild type. Interestingly, the transmission fitness of the MUT-N197 virus was higher than that of the wild type by a factor of 2.78 (95% CI, 1.56, 5.59). However, it is important to note that this estimate is driven heavily by two recipient ferrets, where a large increase in the population of MUT-N197 was observed following transmission from donors infected with a 50:50 ratio of the WT-D197 and MUT-N197 viruses. Although the statistical model is designed to account for the variation among ferrets, we are careful to not draw too strong a conclusion on this result given the limited number of animals in the study.

A large increase in the proportion of MUT virus (>95% of population) was observed following transmission to three recipient ferrets (one in the MUT-Y273 group and two in the MUT-N197 group). To determine if nonsynonymous genetic changes had been acquired across the genome of these viruses, the samples were analyzed by next-generation sequencing. The MUT-Y273 virus in the recipient ferret had acquired a rare T90I HA substitution (contained in only 7 out of 7,373 strains in GISAID), as well as the NP, PA and PB1 genes from the WT-H273 virus, which differed by 4 amino acids compared to the MUT-Y273 virus (see Fig. S1 in the supplemental material). In one ferret, a minor proportion (<20%) of the MUT-N197 virus population contained the NS and PA genes of WT-D197 virus (which differed by 2 amino acids compared to the MUT-N197), but the dominant MUT-N197 viral population from this ferret and from the other did not acquire any amino acid changes following transmission.

## DISCUSSION

This study aimed to characterize two contemporary influenza B variants with either the D197N or H273Y NA substitution. The data showed that the MUT-N197 variant had significantly reduced NA enzyme activity, but the replication and transmission efficiency of this virus in an *in vitro* or *in vivo* model were not notably reduced compared to a closely matched wild-type virus. Further, mathematical analysis from competitive-mixtures experiments found little evidence of reduced relative within-host fitness and no evidence of reduced between-host transmission of the MUT-N197 virus. A previous study using an influenza B virus (B/Rochester/02/2001) with the D197N NA substitution also showed that when ferrets were infected with a mixture of wild-type and variant viruses, a mixture was still maintained at 5 days postinfection (27).

It is interesting that the significantly reduced NA enzyme activity of the MUT-N197 virus did not predict the outcome of the replication or transmission fitness of this variant in the ferret model. This may be because the enzyme-based assay is inherently limited in its ability to capture the complex interplay between the different viral proteins during replication and transmission in a biological setting. This kind of discrepancy between *in vitro* and *in vivo* data has been described previously (35) and highlights the need for caution when assessing viral fitness using only *in vitro* parameters.

As has been seen in other studies, the MUT-Y273 virus had reduced susceptibility to oseltamivir and peramivir but not to zanamivir and laninamivir (36, 37). While the impact on peramivir binding appears to be greater, due to the 85-fold increase in IC_50_ (as opposed to 3-fold for oseltamivir), this is a result of the substantially higher oseltamivir IC_50_ (∼36 nM) compared with the peramivir IC_50_ (∼1 nM) for wild-type influenza B viruses. The actual peramivir and oseltamivir IC_50_s for MUT-Y273 are 80.9 nM and 96.7 nM, respectively, showing that the *in vitro* inhibitory concentrations of the two drugs against the MUT-Y273 virus are similar. We show that the MUT-Y273 virus had NA activity and expression equivalent to those of the WT-H273 virus but delayed growth in cell culture. Previous experiments using an rg-H273Y virus showed that relative to the rg-WT, the rg-H273Y virus had significantly higher NA activity and surface expression and superior replication kinetics in a competitive-mixtures experiment in normal human bronchial epithelial cells (14, 22, 23). The discrepancy in these fitness outcomes between our 2015 H273Y virus and a 1998 rg-H273Y virus may be due to differences in viral background (16 amino acid differences). The MUT-Y273 virus had similar replicative fitness in donor ferrets following intranasal infection viruses and delayed and reduced transmission between cohoused ferrets compared to the WT-H273 virus when tested in a pure population. Genetic analysis showed that the MUT-Y273 virus which transmitted to one of the recipient ferrets contained an M403V NA change, while all of the WT-H273 viruses that infected recipient ferrets contained the L274I PA substitution. Neither of these mutations has been previously reported in the literature to alter viral fitness, and therefore their role in compensating for the NA substation is unknown. Mathematical analysis showed evidence of a slight reduction in the within-host fitness of the variant in a competitive mixture with the wild-type virus but no evidence of reduction in between-host transmission.

There is suggestive evidence that the transmission efficiency of the MUT-Y273 variant was lower when tested as a pure population than when assessed in a competitive-mixtures experiment. It is possible that this difference is due to the reassortment with WT-H273 virus that occurred during the competitive-mixtures experiments, which may have led to an increase in the transmissibility of the MUT-Y273 virus. It is a limitation of this study that while the MUT viruses were closely matched to their paired WT viruses, with identical HA, NA (except for the resistance mutation of interest), and MP sequences, they did have a small number of amino acid differences in the internal genes (Fig. S1). However, these variant viruses represent real circulating strains and therefore have greater relevance than what is obtained from experiments using reverse genetics with laboratory reference strains.

It is important to note that our study contributes to the very limited number of animal studies that have reported influenza B virus transmission to date. One previous study demonstrated successful influenza B transmission between guinea pigs in contact and noncontact models (38), while only two studies (with mixed results) have previously reported on influenza B virus transmission in the ferret model (39, 40). Of these two studies, one study reported successful aerosol and contact transmission of a mouse-adapted influenza B virus, but it could not demonstrate transmission by either model using a nonmouse-adapted isolate (39). The second study using a modified influenza B virus lacking the NB protein showed that one out of the four recipient ferrets was successfully infected by aerosol transmission (40). Our results show successful contact transmission of both the influenza B wild-type viruses tested (4 out of 4 ferrets) and transmission of influenza B variants in the ferret model with circulating human influenza viruses. Given the successful results of our contact ferret transmission model using wild-type and variant strains, future evaluations of the fitness of influenza B strains with reduced NAI susceptibility should consider the use of an aerosol transmission model.

### Conclusion.

The results from our experiments suggest that of two influenza B viruses with reduced NAI susceptibility, the viral fitness of a recent influenza B virus with the H273Y NA substitution was reduced compared to that of an equivalent wild-type virus, but an influenza B virus with a D197N NA substitution showed little reduction in reproduction or transmission in the ferret model. Although the frequency of influenza B viruses with the D197N NA substitution circulating in the community is still less than 1%, it has remained the most commonly reported NAI-resistant strain in recent years. Future studies to determine if the MUT-D197 virus has reduced capacity to transmit via aerosol transmission will be important.

In the future, it is possible that the NA variants examined in our study may gain additional “permissive” mutations that improve their fitness, as was observed with the H275Y NA substitution in seasonal H1N1 viruses (33). This may be more likely for viruses with the D197N NA substitution, which appear to have a smaller fitness deficit than do viruses with the H273Y NA substitution. This knowledge, alongside the fact that clinical reports suggest that oseltamivir is less effective against influenza B and therefore minor changes in the viral NA may further reduce oseltamivir effectiveness, emphasizes the importance of continued monitoring of influenza B viruses for NA substitutions in the future.

## MATERIALS AND METHODS

### Virus selection and stocks.

A B/Yamagata/16/88-like influenza B virus with the D197N NA substitution, designated B/Singapore/GP702/2015 (MUT-N197), was isolated from a 8-year-old female patient not receiving NAI therapy and was submitted to the WHO Collaborating Centre for Reference and Research on Influenza, Melbourne, Australia, by the Ministry of Health, Singapore. Another B/Yamagata/16/88-like virus with the H273Y NA substitution, designated B/Perth/136/2015 (MUT-Y273), was isolated from a 61-year-old female patient not receiving NAI therapy and was submitted during routine surveillance. Wild-type influenza B/Minnesota/23/2015 (WT-D197) and B/England/598/2014 (WT-H273) viruses were kindly provided by the Centers for Disease Control and Prevention (CDC), Atlanta, GA, and the Crick Institute, London, England, respectively, and were specifically chosen as wild-type pairs for MUT-N197 and MUT-Y273, respectively, due to their close genetic homology to the two variant viruses. The WT-D197–MUT-N197 pair had identical gene sequences except for the desired D197N mutation in the NA gene, one amino acid difference in the NS gene, and one amino acid difference in the PA gene Similarly, the WT-H273–MUT-Y273 virus had two amino acid changes in the NP gene, one change in the PB1 gene, and one change in the PA gene (see Fig. S1 for details).

Viruses were propagated in Madin-Darby canine kidney (MDCK) cells, and infectivity titers were determined by calculating the 50% tissue culture infectious dose (TCID_50_/ml), as previously described (41).

### *In vitro* NA inhibition and NA kinetics assay.

To determine the 50% inhibitory concentration (IC_50_) of the influenza B viruses for each NAI, a fluorometric MUNANA [2-(4-methylumbelliferyl)-α-d-*N*-acetylneuraminic acid]-based assay was performed as previously described (42). NAI compounds were purchased from Carbosynth, UK. The Michaelis-Menten constant (*K_m_*; substrate affinity) was also determined by measuring the rate of reaction at different MUNANA concentrations, as previously described (43), but using a MUNANA concentration range from 0 to 800 μM. The IC_50_ was determined and enzyme kinetics assays were performed by three independent experiments.

### Neuraminidase surface expression and activity assay.

Measurement of cell surface NA expression and activity was performed by transfecting HEK-293T (293T) cells with an expression plasmid bearing the NA gene of interest with a C-terminal V5 epitope tag (33, 44, 45).

At 20 h posttransfection, the NA enzyme activity on the cell surface was measured using a modified MUNANA-based assay, as previously described (44). The enzyme expression in these cells was also measured by staining with an anti-V5 Alexa Fluor 647 antibody (Thermo Fisher, Australia) and measuring staining intensity using flow cytometry (44). The NA activity results were normalized for total surface expression, and the results for each variant were normalized against fluorescent staining intensity/activity of the corresponding wild-type viruses. Two independent experiments were done to assess NA expression and activity, where each virus was tested in triplicate.

### *In vitro* replication kinetics.

A multistep replication experiment was carried out in MDCK and MDCK-SIAT1 (SIAT) cell lines, which were grown to confluence in 6-well plates and infected at a multiplicity of infection (MOI) of 0.01 TCID_50_/cell, as described previously (41). Infectivity titers in the samples were then determined using a TCID_50_ assay.

### Ethics statement.

Ferrets (weight range, 600 to 1,500 g) were housed in the Bioresources Facility at the Peter Doherty Institute for Infection. Experiments were conducted with approval from the University of Melbourne Biochemistry & Molecular Biology, Dental Science, Medicine, Microbiology & Immunology, and Surgery Animal Ethics Committee, in accordance with the NHMRC Australian code of practice for the care and use of animals for scientific purposes (project license number 1313040).

### Ferret experiments.

Male and female ferrets age 6 to 12 months old were used in the experiments. All animals received food and water *ad libitum*. All test animals were confirmed to be seronegative to circulating human influenza virus strains using a hemagglutination inhibition assay prior to the experiments. The replication and contact transmission efficiency of the wild-type and variant viruses in the ferret model were tested by experimental infection of ferrets with a “pure” population of wild-type (WT) or mutant (MUT) viruses, as described below. In independent experiments, ferrets were infected with mixtures of wild-type and mutant viruses to determine the relative fitness of each in competitive-mixtures experiments (41, 45, 46). Viral stocks were standardized to 1 × 10^5^ TCID_50_/ml in Dulbecco's phosphate-buffered saline (Sigma, Australia) for ferret inoculation. To make mixtures of WT-H273–MUT-Y273 and WT-D197–MUT-N197 viruses, the standardized stocks were volumetrically mixed at 80:20, 50:50, and 20:80 ratios.

On day 0, donor ferrets were anesthetized by intramuscular injection with 20 mg/ml ilium xylazil-20 (Troy Laboratories, Australia) and intranasally inoculated with 0.5 ml of (i) pure populations of WT (*n* = 4) and MUT (*n* = 4) viruses, or (ii) mixtures at ratios of 50:50 (*n* = 4), 80:20 (*n* = 2), and 20:80 (*n* = 2) of WT and MUT viruses. The ferrets were then housed in high-efficiency particulate air (HEPA)-filtered cages. After 24 h (day 1 postinoculation [p.i.]), one naive recipient ferret was cohoused with one donor ferret to allow for contact transmission. The donor and recipients were cohoused for the entire length of the experiment (11 days). Nasal washes were collected from donors and recipients each day throughout the experiment, and temperature and weight were monitored daily, as described previously (45). All ferrets were euthanized on day 11. Infectious viral load in ferret nasal washes was quantified using a TCID_50_ assay.

### RNA extraction and quantitative analysis of viral RNA in ferret nasal washes.

The viral RNA from ferret nasal wash was extracted and quantified using real-time PCR. The real-time primers were provided by the CDC (Atlanta, USA) and were capable of detecting the influenza B virus NS gene. Internal controls with known RNA copy numbers of B/England/598/2014 virus NS transcripts were generated in-house and included for each assay. This was used to correlate cycle threshold (*C_T_*) with the RNA copy number and quantitate viral RNA in nasal wash samples.

### Molecular analysis of ferret nasal washes.

The proportions of WT and MUT viruses in each nasal wash sample were determined by pyrosequencing (45) using primer pairs specifically designed for PCR amplification and sequencing analysis (Table S3). To validate the performance of the assay, pure populations and known mixtures for each WT-MUT pair were serially diluted and tested across three separate assays. Based on these validation tests, only samples with greater than 4.3 log_10_ NS gene copies/μl were analyzed for mixture estimation.

Next-generation sequencing was completed on nasal wash samples from the last day of viral shedding for each recipient in the pure population study. If the viruses did not transmit, nasal wash samples from the last day of shedding of donors were analyzed. This was done to confirm the genetic stability of the viruses after replication and transmission. In competitive-mixtures experiments, where pyrosequencing results indicated a large increase in MUT viruses in recipient ferrets, nasal wash samples from the last day of viral shedding were also analyzed.

Reverse transcription-PCR (RT-PCR) amplification of all influenza B virus genes was done on extracted viral RNA using universal influenza B virus primers and PCR conditions as described by Zhou et al. (47). Illumina library preparation and sequencing (MiSeq version 2 platform with 150-bp paired-end reads) was done off-site at the Murdoch Children's Research Institute, Melbourne, Australia. The next-generation sequencing (NGS) reads were mapped to their respective WT or MUT genome using Bowtie2 version 2.2.5 (-very-sensitive-local) (http://bowtie-bio.sourceforge.net/bowtie2/index.shtml) after building an index of the references with the bowtie2-build program. SAMtools version 1.7 (https://sourceforge.net/projects/samtools/files/samtools/1.7) was used to process sequence alignments and generate pileup files. The pileup files were then used to build consensus sequence of all genes and scanned for minorities using QUASR version 6.08 (https://sourceforge.net/projects/quasr/) (48).

### Quantitative assessment of differences in virus replication and transmission fitness.

The fitness of a virus depends on its ability to replicate within a host and to transmit between hosts. We used a mechanistic model of viral replication and competition to assess the relative within-host replicative fitness of the variant compared to that of the wild-type virus (45, 49).

As previously described, our model expanded on the standard target cell-infectious cell-virus (TIV) model of viral dynamics (49). The viral population is stratified by strain and accounts for infectious and noninfectious viral material by simultaneously fitting to TCID_50_, real-time PCR, and pyrosequencing data (50).

From the model fit, we estimate the ratio of the basic reproduction number, *R*_0_, between the two viral strains in the competitive mixture, where *R*_0_ describes the expected number of secondary infections produced by a single infected cell in a population of susceptible cells (51).

To assess the relative ability of the variant to transmit between hosts, we fitted a transmission model, as described by McCaw et al., to the pyrosequencing data (46). We utilized the pyrosequencing measurements of the proportion of mutant virus in the donors (on the inferred day of infection) and the recipients. We extended on our previous work to account for pyrosequencing measurement errors in producing our estimates. The transmission model has a single parameter describing the relative transmission fitness of the two strains, where a value greater than 1 indicates enhanced transmissibility of the mutant.

### Statistical analysis.

Viral titers between different groups from the *in vitro* and *in vivo* experiments were analyzed by a two-way analysis of variance (ANOVA) with Bonferroni's *post hoc* analysis using GraphPad Prism 5.0. All other comparisons, such as area under the concentration-time curve (AUC), peak viral titers, NA activity, and *K_m_* values, were made using nonparametric Mann-Whitney's U test. Parameters for the mathematical models used to determine relative finesses (both within and between hosts) were estimated from the data in a Bayesian framework using the R interface to Stan (52, 53) (see Text S4 in the supplemental material).

## Supplementary Material

Supplemental file 1
