# Supplementary material for: Characterization of Influenza B Virus Variants with Reduced Neuraminidase Inhibitor Susceptibility
Source: Antimicrob Agents Chemother. 2018 Oct 24;62(11):e01081-18. doi: 10.1128/AAC.01081-18 (PMC6201084; doi:10.1128/AAC.01081-18)
Supplement: Supplemental file 1 [file zac011187614s1.pdf]

Figure S1

MUT-Y273	NA	HA	NP	MP	NS	PB1	PB2	PA
WT-H273								
NA	H723Y							
HA		0 aa						
NP			V95I,<br>I169V					
MP				0 aa				
NS					0 aa			
PB1						V187I		
PB2							0 aa	
PA								S673G

MUT-N197	NA	HA	NP	MP	NS	PB1	PB2	PA
WT-D197								
NA	D197N							
HA		0 aa						
NP			0 aa					
MP				0 aa				
NS					Y200H			
PB1						0 aa		
PB2							0 aa	
PA								I316L

Figure S1: This figure shows the amino acid differences between each gene from the two different viruses. Sequencing of internal genes were done by extraction of viral RNA using Qiagen RNA extraction kit, amplification of gene segments using primers provided by the WHOCC centre and sanger sequencing using Big Dye Terminator. The sequences were assembled using SeqMan (Lasergene , Australia) and aligned in MegAlign (Lasergene , Australia) using the ClusterW method. Amino acid differences were counted after alignment, and numbering is based on +1 ORF, and all substitution indicate a change from the WT gene.

Figure S2

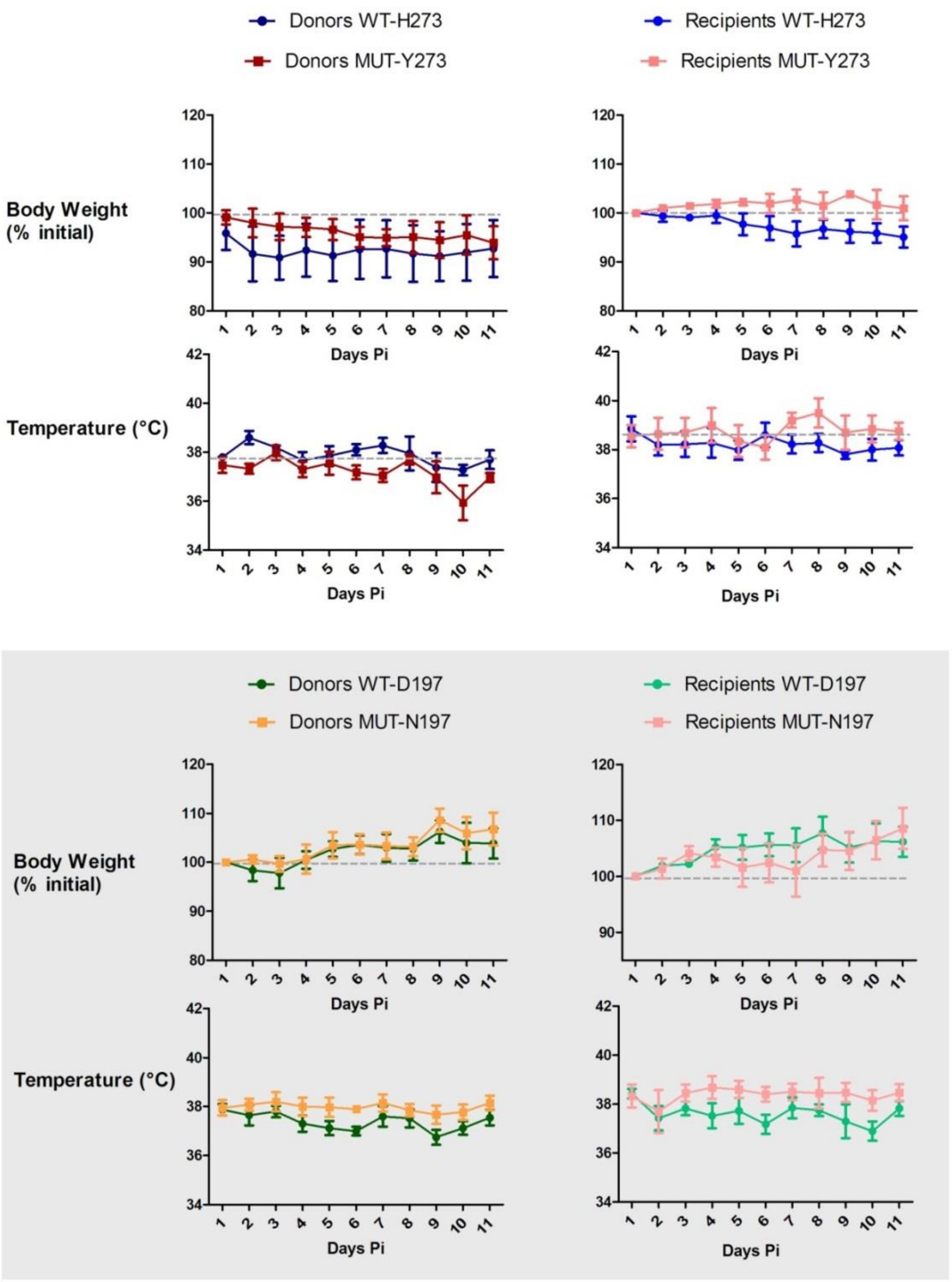

Figure S2: This figure summarizes changes in weight and temperature of ferrets infected with influenza B viruses. Ferrets were experimentally infected with either WT or MUT viruses on Day 0, and monitored for changes weight and temperature to assess clinical symptoms of infections. Experimentally infected ferrets where co-housed with naïve ferrets (recipients) during the length of the experiment (11days) who were also monitored for changes in weight and temperature. The figure summarizes the mean temperature/weight (± SEM) of four ferrets per experimental group.

Table S3

Table S3: Primers used for amplification and sequencing of gene fragments during pyrosequencing

PCR amplification for detecting proportion of H273Y mutation	-5’ Biotin-GGGGAGATTGTTATCTGATGAT 3’
	- 5’GGTCTTTTTGCTGTGTAACGG 3’
Sequencing Primer for Pyrosequencing	5’ CCGCATGTGCATTCCTCAGTAT 3’
PCR amplification for detecting proportion of D197N mutation	5’Biotin-GAGAAGACAGAAACAAGCTGAGGC3’
	5’ CCCGATGCAATTGCAGGC 3’
Sequencing Primer for Pyrosequencing	5’ CTTACATCGGAGTTGATGGCCCA 3’

Text S4

The mathematical models used for the within-host viral dynamics and between-host transmission have been previously described (1-3) .Here we improved the between-host transmission model to allow for observation error in the measurement of the mutant proportion of using pyrosequencing. All inference was performed using the probabilistic programming language STAN(4) accessed via the R interface(5)

The default No U-Turn Sampler (NUTS-HMC) implementation in STAN was used to sample from the posterior distribution for the between-host model. Multiple (4) chains where used to assess convergence. This sampling takes seconds on a standard desktop machine.

Due to prohibitive running times, directly sampling from the posterior distribution of the within-host model was unfeasible (data not shown). Subsequently, we approximated the posterior distribution with a multivariate normal distribution in the constrained space STAN uses to represent parameter space internally. The mode of this distribution and its co-variance matrix were obtained by numerically optimizing the posterior distribution and obtaining the Hessian matrix at this point. It is relatively cheap to sample from this approximation of the posterior distribution. Doing so allowed us to perform the inferences documented in the main text. Due to the large parameter space of the within-host model we used simulated annealing in addition to the built in optimizer to find the maximum a posterior estimate.

The full implementation of the models and an example data set are available at https://github.com/aezarebski/competitive-mixtures. Code used for this publication is at commit afe1d7f35d1d2bf0d769544bb51794eb6367f8b2.

References:

1. **Petrie SM, Butler J, Barr IG, McVernon J, Hurt AC, McCaw JM.** 2015. Quantifying relative within-host replication fitness in influenza virus competition experiments. J Theor Biol **382:**259-271.
2. **Butler J, Hooper KA, Petrie S, Lee R, Maurer-Stroh S, Reh L, Guarnaccia T, Baas C, Xue L, Vitesnik S, Leang SK, McVernon J, Kelso A, Barr IG, McCaw JM, Bloom JD, Hurt AC.** 2014. Estimating the fitness advantage conferred by permissive neuraminidase mutations in recent oseltamivir-resistant A(H1N1)pdm09 influenza viruses. PLoS Pathog **10**.
3. **McCaw JM, Arinaminpathy N, Hurt AC, McVernon J, McLean AR.** 2011. A Mathematical Framework for Estimating Pathogen Transmission Fitness and Inoculum Size Using Data from a Competitive Mixtures Animal Model. PLoS Computational Biology **7:**e1002026.
4. **Carpenter B, Gelman A, Hoffman MD, Lee D, Goodrich B, Betancourt M, Brubaker M, Guo J, Li P, Riddell A.** 2017. Stan: A Probabilistic Programming Language. 2017 **76:**32.
5. **Team SD.** 2017. Rstan: The R interface to Stan. http://mc-stan.org. Accessed
